# Role of Early Trauma on Defense Mechanisms and Coping Strategies in Personality Disorders

**DOI:** 10.3390/medicina61071162

**Published:** 2025-06-26

**Authors:** Fabrizio Turiaco, Fiammetta Iannuzzo, Clara Lombardo, Valentina Clementi, Carmela Mento, Antonio Drago, Antonio Bruno, Maria Rosaria Anna Muscatello, Gianluca Pandolfo

**Affiliations:** 1 Department of Biomedical and Dental Sciences and Morphofunctional Imaging, University of Messina, 98125 Messina, Italy; fabrizio.turiaco@unime.it (F.T.); fiammetta.iannuzzo@unime.it (F.I.); cmento@unime.it (C.M.); maria.muscatello@unime.it (M.R.A.M.); gpandolfo@unime.it (G.P.); 2 Department of “Scienze della Salute”, University of Magna Graecia, 88100 Catanzaro, Italy; clara.lombardo@unime.it; 3 Department of Mental Health, Azienda Sanitaria Provinciale, 89125 Reggio Calabria, Italy; vclementi@hotmail.it; 4 Unit for Psychiatric Research, Psychiatry, Aalborg University Hospital, 9100 Aalborg, Denmark; a.drago@rn.dk

**Keywords:** personality disorders, coping strategies, defense mechanisms, early trauma, mental health

## Abstract

*Background and Objectives*: We investigated whether early psychological trauma can contribute to the development of dysfunctional personality traits and emotional dysregulation. Personality disorders (PDs) are often characterized by using immature defense mechanisms and maladaptive coping strategies. *Materials and Methods*: We evaluated the relationship between early traumatic experiences, PDs, defense mechanisms, and coping strategies. A total of 90 patients aged between 18 and 70 with PDs were studied to detect different subtypes of early trauma, along with the defensive mechanisms and the prevailing coping strategies. Correlation and regression analyses aimed at establishing if specific trauma subtypes were associated with specific defense mechanisms and coping strategies. The Childhood Trauma Questionnaire—Short Form (CTQ-SF), the Defense Style Questionnaire 40 (DSQ-40), and the Coping Orientation to Problem Experienced (COPE) were used as psychodiagnostic tools. *Results*: Our findings determine emotional abuse, emotional neglect, and physical neglect as the most represented subtypes of trauma and underline the use of immature defense mechanisms in PD patients, while adaptive coping strategies, such as social support and positive aptitude were used. *Conclusions*: Early trauma, such as abuse and neglect, can be associated with dysfunctional defense mechanisms and coping strategies. This may have significant implications for managing not only pathological areas of the patient but also the functional ones. Moreover, being aware of these aspects can be useful for psychotherapy, reinforcing therapeutic alliance and reducing symptoms.

## 1. Introduction

Psychological trauma can be defined as any event experienced as extremely stressful, life-threatening, menacing to physical and/or psychological integrity, and overwhelming the individuals’ ability to integrate emotional and cognitive experiences [1]. The impact of trauma is subjective and varies depending on many variables, such as individual differences in emotional and cognitive domains and environmental factors that can be either protective (e.g., the presence of supportive relational networks, stable employment, and financial security) or detrimental. Nevertheless, individual resources may sometimes be inadequate to offer a powerful and adaptive response to stressful events [2], and this is particularly true when traumatic events happen during early life. Early adversity can lead to potentially enduring neurobiological and neuroendocrinological damage, influencing the development of behavioral, physical, socio-emotional, and cognitive domains [3,4].

Early traumatic adversities encompass events, such as the death of a parent, divorce, caregivers’ psychiatric disorders, childhood illnesses, domestic violence, neglect or abandonment, or the absence of emotional support, that may occur during childhood or adolescence [5,6]. Different subtypes of early trauma have been identified and classified: emotional abuse (any behavior that humiliates, embarrasses, frightens, insults, blames, ridicules, or demeans a child); physical abuse (physical aggression that may result in injuries, including hitting or burning; sexual abuse (sexual conduct involving a child, to which the child did not consent or was coerced or manipulated into consenting); emotional neglect (caregiver’s failure to provide the emotional contact and meet a child’s psychological needs, such as providing love, attention, motivation, encouragement, and emotional support); physical neglect (a failure to meet the child’s material needs, including ensuring physical safety, clean and weather-appropriate clothing, proper nutrition, and attention to health needs, such as taking the child to a doctor when necessary).

There is convincing evidence that early childhood traumatic stress significantly contributes to the development of both dysfunctional personality traits and full-fledged psychiatric disorders in adulthood [6], such as depression and anxiety disorders, and suicidal ideation or attempts, the rate of which is from two to five times higher in subjects with early trauma compared to the general population [7,8,9]. Early trauma has also been associated with a more severe course of bipolar disorder [10].

Specific psychopathological trajectories and symptomatic patterns have been associated with distinct subtypes of early trauma: emotional abuse was related to a diminished sense of self-criticism [11], physical abuse to aggressive behaviors, and sexual abuse to sexual dysfunctions [12]. Early trauma can even influence and modulate personality traits, potentially resulting in a predisposition to a sense of guilt, depression, low self-esteem, impaired social skills, repressed anger, hostility, and impulsivity [13,14].

Beginning in the late 1960s, John Bowlby emphasized the importance of adequate caregiving for a child’s development of secure instead of insecure attachment, essential for creating healthy internal working models, namely, mental representations of the self and others [15].

Since the 1990s, several studies have highlighted the association between early trauma and personality disorders (PDs) [16,17,18]. Traumatic experiences impair the capacity to mentalize and symbolize emotions [19], offering a steppingstone for the development of borderline personality disorder (BPD) traits, including affective instability, emotional dysregulation, and self-harming behaviors [20].

Additionally, a “cumulative effect” of traumatic experiences has been described. Children exposed to multiple types of abuse or neglect during development presented more severe borderline personality traits [21]. A systematic review of the last 20 years of literature [22], encompassing 59 studies with over 89,000 patients with PDs, mainly borderline, found an association between the development of the disorder and the presence of at least one childhood trauma, as confirmed by 52 studies. Specifically, emotional abuse and sexual abuse ranked as the first and second most significant predictors, followed by physical abuse, emotional neglect, and, finally, physical neglect.

Analogously, Grecucci and collaborators [23] have demonstrated that those individuals whose childhood was characterized by emotional neglect or physical abuse showed alterations in the so-called “impulsivity network,” formed by prefrontal regions, such as the inferior frontal gyrus, insula, and the postcentral and precentral gyri. Conversely, individuals who experienced physical neglect exhibited impairments in the “interpersonal network,” including the temporoparietal junction and smaller regions within the anterior and posterior midline structures and the insula.

The mechanisms by which people deal with perceived threats, harm, and loss can be unconscious patterns aimed at facing distress and conflicts through a more or less severe reality distortion (defense mechanisms) and partially conscious coping strategies, which are attempts at managing unpleasant emotions and general stressors operating at a lesser degree of reality distortion [24,25].

Studies on defense mechanisms [26] strongly supported their role in personality structure evaluation. A vast body of scientific literature has integrated the description of personality organization and functioning, considering prevailing defensive patterns. Defense mechanisms can be described as mature when they provide the individual with a greater ability to adapt to internal and external conflicts without significant reality distortion. Conversely, immature defense mechanisms appear during the first developmental phases, imply a higher reality distortion, are less effective in adjustment, and have been associated with various forms and degrees of psychopathology [27]. An example of immature defense is “projection,” a mechanism that protects the individual from self-awareness of devalued, unacceptable internal parts by transferring them onto other people, with the consequence of a serious reality distortion expressed by suspiciousness and paranoia within the context of neuroticism, which reflects tendencies toward negative affective states, such as fear, sadness, and distress. Generally, in these models, mature defenses are associated with adaptive functioning [28], whereas neurotic and immature mechanisms are linked to maladaptive functioning or lower conscientiousness. Furthermore, research has shown that an overwhelming use of immature defenses is related to depression and anxiety, dysfunctional personality traits (e.g., borderline, dependent, avoidant, narcissistic, and antisocial traits), and an increased risk of suicide [29,30,31].

Coping strategies are commonly classified into problem-focused and emotion-focused coping [32,33]. Problem-focused coping targets the stressor itself and includes efforts to remove, avoid, or mitigate its impact if unavoidable. Emotion-focused coping, alternatively, reduces negative emotional states and distress, either by seeking emotional support in the environment, the overt expression of negative emotions (e.g., shouting or crying), positive reappraisal, or distraction. Although both approaches can be helpful for managing stressors and adversity, emotion-focused coping is not generally as adaptive as problem-focused coping. Coping strategies tend to remain stable over time in response to various stressors, and they are presumably influenced by personality structure, which itself is shaped by genetic vulnerability, temperament, and environmental factors [34]. A review [35] aimed at analyzing studies on possible associations between specific personality traits and coping strategies showed that extraversion, a personality dimension characterized by ease in approaching others, seeking positive emotions, sociability, assertiveness, and high energy, with a strong sensitivity to rewards, was related to engagement or approach coping, which encompasses problem-focused coping along with emotion-focused coping (support seeking, cognitive restructuring, and problem solving). Contrarily, neuroticism was associated with the use of disengagement coping, a form of emotion-focused coping based on denial, avoidance, and wishful thinking.

There is solid evidence connecting childhood trauma, neglect, and dysfunctional personality with the development of maladaptive coping mechanisms [36,37,38]. Research shows that individuals with PDs tend to resort to dysfunctional coping styles, such as mental disengagement, social isolation, excessive self-blame, and/or blaming others [39]. Main results from studies in this area of research are reported in Table 1.

Based on the existing evidence, the relationship among early traumatic experiences, PDs, defense mechanisms, and coping strategies deserves further investigation. The aim of this study was to evaluate the possible influence of early trauma on defense mechanisms and coping strategies in a sample of patients with PDs, starting from the assumption that early adversities may affect emotional and cognitive development, personality traits, emotional regulation, and adaptive responses. Previous studies have rarely addressed the joint study of defense mechanisms and coping strategies. Considering the complementary role of both processes in adjustment to multiple exposures to adeverse childhood experiences (ACEs) [12], more research is needed on the topic. Moreover, the study of the relationship between two core features of different approaches may provide some evidence, to some extent, in favor of the underlying mechanisms for psychotherapy integration. Different from previous studies, our research provided a concurrent, joint assessment of defense mechanisms and coping strategies, two main psychological constructs with a complementary role in adjustment to exposure to early traumatic events and in managing emotional distress and stressful experiences.

## 2. Materials and Methods

### 2.1. Subjects

Inpatients and outpatients, aged between 18 and 70 years, referred to the Psychiatry Unit of the University Hospital (omitted for blinded version) and to the Psychiatric Residential Clinic (omitted for blinded version), with a DSM-5-TR [47] previous diagnosis of “Unspecified Personality Disorders” made by a psychiatrist with at least 5 years of experience, were consecutively included in the study using the recruitment method applied in our previous research on PDs [48]: the selection procedures, the inclusion and exclusion criteria, the Institutional Review Board Statement, and the Informed Consent Statement can be consulted in the specific “Materials and Methods” section. The diagnosis of PD without other specification refers to the framework of mixed personality disorder: a stable pattern of altered internal experience and behavior, pervasive in multiple areas of functioning of the individual, which includes the characteristics of several personality disorders without meeting the criteria for a specific one [49]. Omitting this construct would possibly lead to a diagnostic underestimation for all those patients affected by PD but who do not meet the criteria for the diagnosis of a specific PD [50]. Furthermore, it should be considered that the diagnosis of PD not otherwise specified accounts for an estimated prevalence rate of 3–6% in the general population [51].

### 2.2. Measures

The following instruments were used:

Childhood Trauma Questionnaire—Short Form (CTQ-SF)—Italian version

The CTQ-SF is a self-report tool that investigates traumatic experiences in childhood, and it is formed by 28 items, rated on a five-point Likert scale, ranging from “never true” to “very often true.” Scores for each clinical scale range from 5 (absence of traumatic events) to 25 (severe history of abuse). The tool provides a total score and five subscale scores corresponding to the following dimensions: “Emotional Abuse”: evaluates verbal aggression undermining a child’s self-esteem and well-being; “Physical Abuse”: assesses physical aggression by an adult resulting in harm or injury to the child; “Sexual Abuse”: examines any sexual conduct involving a non-consenting child and an adult; “Emotional Neglect”: reflects a caregiver’s failure to meet a child’s basic psychological needs, such as love, support, and a sense of belonging; “Physical Neglect”: identifies a caregiver’s failure to fulfill a child’s basic physical needs, including nutrition, safety, protection, supervision, and health care. The Italian translation presents a good reliability (Cronbach’s alpha) across all scales (Emotional Abuse = 0.88, Physical Abuse = 0.95, Sexual Abuse = 0.96, Emotional Neglect = 0.91, and Physical Neglect = 0.87) [52]. In our sample, the total Cronbach’s alpha was estimated at 0.71.

Defense Style Questionnaire 40 (DSQ-40)—Italian version

This self-report tool is constituted by 40 items and assesses individual defensive functioning across 20 defense mechanisms (two items per mechanism). The defense mechanisms are classified into three styles: “Mature Style” (8 items): sublimation, humor, anticipation, and suppression; “Neurotic Style” (8 items): undoing, pseudo-altruism, idealization, and reactive formation; “Immature Style” (24 items): projection, passive aggression, acting out, isolation, devaluation, autistic fantasy, denial, displacement, dissociation, splitting, rationalization, and somatization. Participants rank each item on a nine-point Likert scale ranging from 1 (“strongly disagree”) to 9 (“strongly agree”). Scores for individual defense mechanisms are calculated as the mean of the two items measuring each defense. Scores for defense styles are averaged across the items within each style, resulting in scores ranging from 1 to 9, with higher scores indicating greater use of the corresponding defense mechanism or style. The Italian version presents a sufficient reliability (Cronbach’s alpha) across the three styles (Mature Style = 0.61, Neurotic Style = 0.59, Immature Style = 0.80) [53]. In our sample, the total Cronbach’s alpha was estimated at 0.87.

Coping Orientation to Problem Experienced—New Italian Version (COPE-NVI)

COPE-NVI is a tool consisting of 60 statements, with responses rated on a four-point Likert scale from “I usually do not do this at all” to “I do this almost always.” The total score ranges from 60 to 240, with higher scores indicating greater psychological well-being in stressful situations. The tool leads participants to respond based on their usual approach to stressful situations rather than a specific event, assessing five dimensions: “Social Support”: seeking understanding, information, and emotional expression; “Avoidance Strategies”: employing denial, behavioral disengagement, and mental disengagement; “Positive Attitude”: adopting acceptance and positive reinterpretation of events; “Problem Orientation”: employing active, positive strategies, including planning; “Transcendent Orientation”: engaging in religious practices and a lack of humor. The Italian translation presents a good reliability (Cronbach’s alpha) across the five dimensions (Social Support = 0.91, Avoidance Strategies = 0.70, Positive Attitude = 0.76, Problem Orientation = 0.83, and Transcendent Orientation = 0.85) [54]. In our sample, the total Cronbach’s alpha was estimated at 0.76.

### 2.3. Statistical Analysis

Only fully complete questionnaires were considered for statistical analysis. Descriptive statistics (mean ± standard deviation (SD); frequency and percentages) were used to summarize continuous and non-continuous demographic and psychometric data, as requested. A correlation analysis was performed to evaluate possible associations between early trauma, defense mechanisms, and coping strategies. Further, all the variables that reached statistical significance in the correlation analyses were entered into five linear regression models (DSQ-40 “Mature” and “Immature” styles; COPE-NVI “Social Support,” “Positive Attitude,” and “Problem Orientation” dimensions—as dependent variables; and CTQ-SF “Emotional Abuse,” “Emotional Neglect,” and “Physical Neglect” subscales—as independent variables) in order to investigate which early trauma was a predictor of defense mechanisms and coping strategies. Data were analyzed with IBM SPSS Statistics 23.0 (IBM Corp: Armonk, NY, USA). A *p*-value of < 0.05 was considered statistically significant.

## 3. Results

Of the 131 eligible subjects, 17 refused to participate and 24 were excluded based on the exclusion criteria or due to incomplete questionnaires. The final sample consisted of 90 patients (57.8% female), with a mean age of 41.4 years (±12.7 S.D.). Educational attainment was distributed as follows: 38.9% held a lower secondary school diploma, 36.7% an upper secondary school diploma, and 15.6% a university degree. A total of 29 subjects (31.8%) presented comorbidity with depressive disorders, 24 (27.4%) with anxiety disorders, 16 (17.9%) with bipolar disorder, 13 (14.4%) with psychotic disorders, and 8 (8.5%) with eating disorders; furthermore, at the time of the study, 82 subjects (91%) were taking psychopharmacological polytherapy, and only 8 patients (9%) were on monotherapy. Regarding personality traits, borderline-type traits were prevalent in 42% of subjects, followed by avoidant (21%), narcissistic (18%), and schizoid (8%) types.

Table 2 presents the mean scores for the administered clinical scales in the total sample. For the CTQ-SF, descriptive statistics revealed moderate levels of “Emotional Neglect” (mean ± S.D. = 16.28 ± 5.35), “Physical Neglect” (mean ± S.D. = 12.20 ± 2.56), and “Emotional Abuse” (mean ± S.D. = 11.41 ± 5.90); low levels were observed for “Physical Abuse” (mean = ± S.D. = 8.76 ± 1.06) and “Sexual Abuse” (mean ± S.D. = 7.27 ± 5.08). Of the total sample, 76 subjects (84.4%) reported clinically significant scores for “Physical Neglect,” 55 (61.1%) for “Emotional Neglect,” 38 (42.2%) for “Physical Abuse,” 29 (32.2%) for “Emotional Abuse,” and 18 (20%) for “Sexual Abuse.” Concerning the DSQ-40, a higher prevalence of “Immature Style” (mean ± S.D. ± S.D. = 4.02 ± 1.25) was observed, while “Mature Style” (mean ± S.D. = 4.08 ± 1.37) and “Neurotic Style” (mean ± S.D. = 4.36 ± 1.61) were below their respective clinical cut-off thresholds. Regarding the COPE-NVI, the most frequently utilized coping strategies were “Social Support” (mean ± S.D. = 31.21 ± 8.25) and “Positive Attitude” (mean ± S.D. = 29.24 ± 7.07).

Table 3 reports the correlation analyses carried out to evaluate the possible associations between early traumatic experiences (as measured by the CTQ-SF scales), coping strategies (COPE-NVI), and defensive mechanisms (DSQ-40): the data highlighted a significant positive association between “Emotional Abuse” and the DSQ-40 style “Immature” (*p* = 0.014), a significant positive association between “Emotional Neglect,” the COPE-NVI dimensions “Positive Attitude” (*p* = 0.006) and “Problem Orientation” (*p* = 0.036), and the DSQ-40 style “Mature” (*p* = 0.018), and a significant positive association between “Physical Neglect” and the COPE-NVI dimension “Social Support” (*p* = 0.011).

Further, all the variables that reached statistical significance in the correlation analyses were entered into five linear regression models (DSQ-40 “Mature” and “Immature” styles; COPE-NVI “Social Support,” “Positive Attitude,” and “Problem Orientation” dimensions—as dependent variables; and CTQ-SF “Emotional Abuse,” “Emotional Neglect,” and “Physical Neglect” subscales—as independent variables) in order to investigate which early trauma was a predictor of defense mechanisms and coping strategies (Table 4). The results indicate that the CTQ-SF “Emotional Neglect” and “Physical Neglect” subscales were direct predictors of the COPE-NVI domains “Positive Attitude” (*p* = 0.017) and “Social Support” (*p* = 0.038), respectively. Conversely, early trauma did not significantly contribute to the prediction of defense mechanisms.

## 4. Discussion

Our study was aimed at evaluating the relationships among early trauma, defense mechanisms, and coping styles in patients with PDs; to the best of our knowledge, this is the first study in the scientific literature specifically investigating this relationship in PDs.

In our sample, all subtypes of early trauma were present, with scores over the normal range; however, the most represented subtypes were emotional abuse, emotional neglect, and physical neglect. Scores on the dimensions of physical abuse and sexual abuse were slightly over the cut-off. Our findings are congruent with previous studies on the role of childhood trauma as a central facet of personality development, although few specific associations between trauma subtypes and PDs have been found, except for BPD [20].

As for defense mechanisms, our sample was characterized by the prevalent use of immature defenses, as assessed by DSQ-40 mean scores that fell marginally above the cut-off. Immature defense mechanisms encompass projection, passive aggression, acting out, affect isolation, devaluation, schizoid fantasy, denial, displacement, dissociation, rationalization, splitting, and somatization; as they reduce the conscious experience of negative emotions by a gross distortion of interpersonal reality, immature defenses have been found to be associated with lower psychosocial functioning and are most prevalent in PDs [55].

Conversely, when examining coping results, our sample displayed scores within the normal range, with a tendency to use more frequently the social support and positive attitude strategies. Although research on this topic reports mixed results, it is quite accepted that patients exhibiting personality disorder are characterized by maladaptive coping strategies, usually including self- or other-blame, catastrophizing, and rumination. Accordingly, it has been suggested that seeking social support as a coping strategy may be less available to subjects with PDs, who usually display socio-relational difficulties, with fewer and less functional relationships [56]. This is in contrast with the observed tendency of our sample to rely on social support; nevertheless, the finding is congruent with the results of a previous study that has shown the prevalence of this coping mechanism in patients with BPD comorbid with ADHD [57].

When analyzing possible correlations among early trauma, defense mechanisms, and coping strategies, we found that emotional abuse was associated with immature defense mechanisms, whereas no significant correlations with coping strategies were found. These findings are barely comparable with data from the recent literature, for the main reason that the predominant use of immature defenses and avoidant coping strategies has been found in affective disorders, also comorbid with PDs [58,59]. In a sample of depressed patients, immature defenses were more represented in those with comorbid PDs; moreover, immature defenses were reliable predictors of worse outcomes in psychotherapy at a 6-month follow-up [60]. Emotional neglect was associated with mature defenses, which include anticipation, sublimation, suppression, altruism, and humor, and with functional coping strategies, such as positive attitude and problem orientation. These results were quite unexpected, in that mature defenses imply the abilities of self-reflection and reality testing; positive attitude can be described as the aptitude for keeping motivation and confidence in challenging situations, whereas problem orientation is the ability to target useful elements for problem-solving; thus, they are considered functional coping strategies. As previously mentioned, PDs are usually expected to be related to immature defenses and maladaptive or dysfunctional coping strategies; although few studies have empirically investigated the reciprocal influences of coping mechanisms and PDs, main results pointed at the presence of strategies based on avoidance, behavioral passivity, uncontrolled discharge of emotions, and mental disengagement at the expense of problem-focused, active coping, and social support seeking [38,61].

In our sample, the positive relation among emotional neglect, mature defenses, and functional coping strategies (positive attitude) could be explained by recurring to the “early adultification” model [62]. Emotional neglect, which involves caregivers’ failures in addressing a child’s emotional and developmental needs and in providing emotional support and validation, can result in the assumption of premature self-sufficiency and adult-like roles, including caregiving. Thus, adultified children may develop strong practical life skill competence and early independence, but they could still struggle with negative emotions, such as anxiety, depressive tendencies, and worries. Yet, as a consequence of early adultification, very different long-term outcomes in emerging adulthood may be expected, ranging from negative psychopathological features, such as internalizing symptoms, risk-taking behaviors, and poor competency in close relationships, to more adapted and functional behaviors characterized by increased self-confidence, a sense of agency, and positive parent–child relationships [63].

Physical neglect, not related to defense mechanisms, was positively associated with coping strategies based on social support seeking, reflecting a reliance on others for care and support. These findings are barely comparable with the existing literature, since no studies have specifically evaluated the potential association of physical neglect with specific coping and/or defense mechanisms in PDs. In the general population, in a sample of young adults, research on the potential impact of emotional and physical subtypes of neglect on mental health outcomes has shown that only emotional neglect was a risk factor for psychopathological syndromes, such as depression, stress, and anxiety, whereas physical neglect was not associated with any mental health outcome [64]. It can be hypothesized that physical neglect may have a mild and less pronounced pathoplastic role, since it is presumably the most detectable early trauma subtype, when considering that it consists of noticeable physical consequences, such as poor hygiene, malnutrition, and lack of medical care. In this case, functional coping strategies, such as social support seeking, as found in our sample, could be read as a compensatory strategy aimed at facing this kind of early adversity.

Finally, no significant correlations were found among physical and sexual abuse, defense mechanisms, and coping strategies, in partial alignment with the literature, which mainly focused on other symptoms, such as dissociative phenomena [65] or sexual dysfunction in survivors of sexual abuse [66,67], which correlated to avoidance-based coping strategies. On the other hand, data on physical abuse and its potential impact on mental health trajectories and/or specific syndromes are sparse, in that only a few studies have separately evaluated this early trauma subtype and its associations with psychopathology [68,69,70].

### Limitations

The findings of the present study, although providing insight on several psychopathological dimensions, such as early trauma subtypes, defense mechanisms, and coping patterns, not always related to the classic nosography of PDs, need to be cautiously interpreted according to a number of limitations that encompass the small sample size and the diagnosis of Unspecified Personality Disorder that hinder the possibility of investigating possible differences among single PDs. Furthermore, it should be borne in mind that self-report measures are potentially subject to biases, especially in a population whose self-perception may be affected by the very processes under study. Future research should enroll a larger group of patients and focus on specific PDs to better evaluate how much specific characteristics affect the correlations sought by our study.

## 5. Conclusions

This study focused on early adverse experiences, unconscious (defense mechanisms), and conscious (coping) functioning in a sample of patients exhibiting personality disorder. Early trauma, such as abuse and neglect, can be associated with dysfunctional defense mechanisms and coping strategies. These particular areas of functioning may have significant implications for treatment, starting from the pervasive and long-lasting impact of childhood adversities, along with the available adjustment strategies, on personality development. Both defenses and coping styles are not only contributing factors to the course of PDs; they should also be considered as psychological and behavioral dimensions that can shape the areas of vulnerability, as well as the functional areas of the patient. In psychotherapeutic treatment, addressing these individual features can build or reinforce therapeutic alliance, contributing to symptom reduction and, mainly, leading to new understandings and improved psychodynamic trajectories.

## Figures and Tables

**Table 1:** Main studies about defense mechanisms, coping strategies, and personality disorders.

References	Type of Study	Number of Participants	Main Findings
Carlson et al., 2020 [40]	Clinical trial	N = 233; BPD	Social diversion-oriented coping may improve multiple facets of functioning.
Aguglia et al., 2024 [41]	Clinical trial	N = 224; BPD	Hopelessness was significantly associated with female gender, suicidal ideation, number of suicide attempts, medication and alcohol misuse, reduced ability to cope with stressors, and alexithymia.
Asgarizadeh et al., 2023 [42]	Clinical trial	N = 600; general population; BPD traits	The study identified maladaptive clusters of shame-coping independent from emotion regulation ability yet related to personality pathology, mentalizing deficits, and disordered attachment.
Chauhan et al., 2023 [43]	Clinical trial	N = 107; BPD	Dysfunctional coping skills were associated with psychological distress at the 9-month follow-up and rumination at the 1-year follow-up.
Wang et al., 2023 [12]	Clinical trial	N = 1629; MDD	Personality dimensions were associated with physical neglect and emotional abuse. All types of child maltreatment (excluding sex abuse) showed an association with coping styles.
Lee et al., 2020 [31]	Clinical trial	N = 125; BPD	Suicide attempters were characterized by self-sacrificing splitting and affiliation defense styles.
Carvalho et al., 2019 [44]	Clinical trial	N = 320; general population	Pathological personality traits were associated with immature defense mechanisms.
Yun et al., 2024 [45]	Clinical trial	N = 227; BPD	Prevalence of maladaptive and image-distorting in BPD.
Euler et al., 2025 [46]	Clinical trial	N = 60; BPD	After 6 months, but not 12 months, of DBT, patients with minor image-distorting defenses showed significant reduction in self-harm.

BPD: Borderline Personality Disorder; MDD: Major Depressive Disorder; DBT: Dialectical Behavior Therapy.

**Table 2:** Descriptive statistics (mean ± SD) for the total sample (n = 90).

Scales	Minimum	Maximum	Mean	SD	Cut-Off/Range
CTQ-SF					
Physical abuse	5	25	8.76	5.29	≥8
Sexual abuse	5	25	7.27	5.08	≥6
Emotional abuse	5	25	11.41	5.90	≥9
Emotional neglect	5	25	16.28	5.35	≥8
Physical neglect	5	18	12.20	2.58	≥10
DSQ-40					
Mature Style	1.00	7.37	4.08	1.37	≥5.4
Neurotic Style	1.37	7.75	4.36	1.61	≥4.5
Immature Style	0.83	6.80	4.02	1.25	≥3.6
COPE-NVI					
Social Support	15	48	31.21	8.25	12–48
Avoidance Strategies	18	49	31.06	7.11	16–64
Positive Attitude	14	42	29.24	7.07	12–48
Problem Orientation	12	46	28.30	7.78	12–48
Transcendental Orientation	11	32	21.78	5.07	8–32

**Table 3:** Correlation analysis.

CTQ-SF	DSQ-40			COPE-NVI				
	Mature	Neurotic	Immature	Social Support	Avoidance Strategies	Positive Attitude	Problem Orientation	Transcendent Orientation
Physical Abuse	0.061	0.026	0.116	0.095	0.073	0.118	0.173	0.202
Sexual Abuse	−0.166	0.191	0.111	−0.003	0.014	−0.153	−0.130	0.081
Emotional Abuse	−0.100	0.140	0.259 *	0.093	0.033	−0.062	−0.016	−0.005
Emotional Neglect	0.249 *	0.065	−0.164	0.146	0.026	0.290 **	0.221 *	0.127
Physical Neglect	0.109	0.173	0.102	0.267 *	0.127	0.202	0.192	0.080

* *p* < 0.05; ** *p* = 0.006.

**Table 4:** Linear regression analysis.

		Unstandardized Coefficients		Standardized Coefficients		
Dependent Variable	Predictors	B	S.E.	Beta	t	*p*
Mature Style ^a^ (Model 1)	(Constant)	2.738	0.878		3.117	0.002
	Emotional abuse	0.004	0.028	0.016	0.137	0.892
	Emotional neglect	0.063	0.032	0.244	1.976	0.051
	Physical neglect	0.023	0.058	0.044	0.402	0.689
Immature Style ^b^ (Model 2)	(Constant)	3.084	0.795		3.879	0.000
	Emotional abuse	0.046	0.025	0.215	1.826	0.071
	Emotional neglect	−0.023	0.029	−0.099	−0.808	0.421
	Physical neglect	0.065	0.052	0.133	1.232	0.221
Social Support ^c^ (Model 3)	(Constant)	15.353	5.190		2.958	0.004
	Emotional abuse	0.247	0.163	0.177	1.514	0.134
	Emotional neglect	0.260	0.187	0.169	1.393	0.167
	Physical neglect	0.721	0.342	0.226	2.109	0.038
Positive Attitude ^d^ (Model 4)	(Constant)	17.678	4.439		3.983	0.000
	Emotional abuse	0.094	0.140	0.078	0.670	0.504
	Emotional neglect	0.388	0.160	0.293	2.426	0.017
	Physical neglect	0.343	0.293	0.125	1.173	0.244
Problem Orientation ^e^ (Model 5)	(Constant)	16.577	4.969		3.336	0.001
	Emotional abuse	0.125	0.156	0.094	0.797	0.427
	Emotional neglect	0.335	0.179	0.230	1.870	0.065
	Physical neglect	0.398	0.327	0.132	1.215	0.228

^a^ R = 0.253, R-squared adjusted = 0.031, F = 1.957, *p* = 0.126; ^b^ R = 0.292, R-squared adjusted = 0.053, F = 2.676, *p* = 0.052; ^c^ R = 0.318, R-squared adjusted = 0.070, F = 3.232, *p* = 0.026; ^d^ R = 0.325, R-squared adjusted = 0.074, F = 3.382, *p* = 0.022; ^e^ R = 0.273, R-squared adjusted = 0.042, F = 2.308, *p* = 0.082.

## Data Availability

The datasets used and/or analyzed during the current study are available from the corresponding author on reasonable request.
